# Optimizing near Infrared Reflectance Spectroscopy to Predict Nutritional Quality of Chickpea Straw for Livestock Feeding

**DOI:** 10.3390/ani11123409

**Published:** 2021-11-29

**Authors:** Tena Alemu, Jane Wamatu, Adugna Tolera, Mohammed Beyan, Million Eshete, Ashraf Alkhtib, Barbara Rischkowsky

**Affiliations:** 1 Department of Animal and Range Sciences, Hawassa College of Agriculture, Hawassa University, Hawassa P.O. Box 5, Ethiopia; atolera@yahoo.com (A.T.); mohadbeyan@yahoo.com (M.B.); 2 International Centre for Agricultural Research in Dry Areas, Addis Ababa P.O. Box 5689, Ethiopia; J.Wamatu@cgiar.org (J.W.); b.rischkowsky@cgiar.org (B.R.); 3 Department of Plant Science/Plant Breeding, Ethiopian Institute of Agricultural Research, Addis Ababa P.O. Box 2003, Ethiopia; million102001@yahoo.com; 4 School of Animal, Rural and Environmental Sciences, Nottingham Trent University, Brackenhurst Campus, Southwell, Nottinghamshire NG25 0QF, UK; a.s.alkhtib@gmail.com

**Keywords:** calibration, validation, prediction error, nutritional quality, crop residue, NIRS

## Abstract

**Simple Summary:**

The potential of near infrared reflectance spectroscopy (NIRS) to predict the nutritive value of chickpea straw was identified. Spectral data of 480 samples of chickpea straw (40 genotypes) were scanned with a spectral range of 1108 to 2492 nm. The samples were reduced to 190 representative samples based on the spectral data then divided into a calibration set (160 samples) and a cross-validation set (30 samples). All 190 samples were analysed for dry matter, ash, crude protein, neutral detergent fibre, acid detergent fibre, acid detergent lignin, Zn, Mn, Ca, Mg, Fe, P, and in vitro gas production metabolizable energy using conventional methods. The prediction equations were generated by multiple regression analysis. The NIRS prediction equations in the study accurately predicted the nutritive value of chickpea straw (R^2^ of cross validation > 0.68; standard error of prediction < 1%). Chickpea straw nutritive value could be predicted using NIRS.

**Abstract:**

Multidimensional improvement programs of chickpea require screening of a large number of genotypes for straw nutritive value. The ability of near infrared reflectance spectroscopy (NIRS) to determine the nutritive value of chickpea straw was identified in the current study. A total of 480 samples of chickpea straw representing a nation-wide range of environments and genotypic diversity (40 genotypes) were scanned at a spectral range of 1108 to 2492 nm. The samples were reduced to 190 representative samples based on the spectral data then divided into a calibration set (160 samples) and a cross-validation set (30 samples). All 190 samples were analysed for dry matter, ash, crude protein, neutral detergent fibre, acid detergent fibre, acid detergent lignin, Zn, Mn, Ca, Mg, Fe, P, and in vitro gas production metabolizable energy using conventional methods. Multiple regression analysis was used to build the prediction equations. The prediction equation generated by the study accurately predicted the nutritive value of chickpea straw (R^2^ of cross validation > 0.68; standard error of prediction < 1%). Breeding programs targeting improving food-feed traits of chickpea could use NIRS as a fast, cheap, and reliable tool to screen genotypes for straw nutritional quality.

## 1. Introduction

Chickpea is one of the key pulses in the world [1]. Cultivated chickpeas are categorized into two main groups: Desi and Kabuli [1]. Desi grains are small, dark in colour, and smooth or wrinkled and are preferred for use as flour [1]; however, they are used for direct cooking. Kabuli grains are large and cream in colour and contain less fibre than Desi grains. Thus, Kabuli grains are used for whole grain cooking [1]. The world yield of chickpea grains was 14,246,000 t in 2019, which accounts for 12% of the world pulse grain. Chickpea grains have high levels of protein, minerals, and vitamins for human consumption [1]. Moreover, growing chickpea improves soil fertility, land use intensity, and provides households with cash supply [2]. It has been reported that the production of 1 kg of chickpea grains is associated with production of 1.55 kg of straw [3]. Accordingly, chickpea cultivation generates large amounts of straw (~221,001,000 t), which is used for ruminant feeding. Chickpea straw contains 65 g/kg of crude protein (CP), 694 g/kg of neutral detergent fibre (NDF), 516 g/kg of acid detergent fibre, 111 g/kg of acid detergent lignin, and 7.7 MJ/kg of metabolizable energy [1]. Chickpea straw has a high content of antinutritional factors like tannins and oxalates [1].Yet, ruminant animals were reported to have a high tolerance to tannins and oxalates if the diet was adequately supplemented with protein and energy [1]. Chickpea straw is also used as soil mulch in mixed farming systems [4]. Accordingly, leveraging grain yield alongside straw yield and nutritive value will improve the biomass supply for human and livestock consumption and will trigger greater use of cereal straw for soil mulching [4]. Varietal selection to increase the yield and nutritive value of chickpea straw is promising [3,5,6]. The improvement programme of chickpea leaded by the International Centre of Agricultural Research in the Dry Areas (ICARDA) and national research centres in developing countries started recently to recognize the importance of chickpea straw’s nutritive value and started to include it as a selection criterion in their breeding programs. These programs are expected to produce large sets of chickpea straw samples to be analysed for nutritional value [7]. The conventional methods of feed evaluation cannot cope with this huge number of samples since they are expensive and time consuming. The near infrared spectroscopy (NIRS) technique has been applied for the nutritional characteristics of animal feeds [8,9]. Unlike most conventional analytical methods, the NIRS technique is fast, low-cost, and nondestructive to the sample. Additionally, NIRS requires very little sample preparation and no chemicals [10]. It is consistent, accurate, and fast [10]. Furthermore, NIRS can be used to analyse multiple feed nutrient properties at one time [8]. Generally used as a quantitative and qualitative analysis method, NIRS technology requires the development of prediction models, which involves multivariate analysis and analytical chemistry to extract the most relevant information [10]. The ability of NIRS technology to determine the nutritive value of chickpea straw was evaluated by Dereje et al. [11]. Yet, the application of the results of this study is limited because the number of chickpea samples was marginal and the genotypic and locational characters of the samples were unknown.

Developing NIRS equations for chickpea straw nutritional analysis would facilitate the process of chickpea improvement for food and feed production, which would promote sustainable food production in mixed farming systems. Although the reliability of NIRS has been well investigated for temperate feeds, no studies have reported on the feasibility of the use of NIRS to determine the nutritive value of chickpea straw. Thus, the goal of the current study is to determine the accuracy and robustness of NIRS to determine the nutritive value of chickpea straw for screening the genotype in multi-dimensional improvement programs.

## 2. Materials and Methods

### 2.1. Sample Description and Experimental Layout

A total of 480 samples of chickpea straw from preliminary and national variety trials were used in the current study. The description of the sampling areas is presented in Table 1. The straw samples were collected after harvest, naturally dried for 7 days, and then stored in paper bags until they were analysed.

### 2.2. Spectral Analysis of Chickpea Straw Samples

All samples were ground (1 mm sieve size), dried at 60 °C overnight in an oven to standardize the moisture content, and then scanned using Foss NIRS 5000 with software package WinISI II in a spectral range of 1108 to 2492 nm (Win Scan version 1.5, 2000, intrasoft international, L.L.C, Luxembourg). Optical values were recorded as log 1/Reflectance.

### 2.3. Chemical Analysis Using Conventional Methods

In total, 190 samples were subsampled out of the 480 samples using the CENTRE algorithm based on NIRS spectra data [12]. The samples were analysed for dry matter (DM), ash, and crude protein (CP) according to the methodology of AOAC [13]. The sample was ashed in a muffle furnace at 500 °C overnight (method 942.05) to determine the ash content. The Kjeldahl method using Kjeldahl (protein/nitrogen) Model 1026 (Foss Technology Corp., Hilleroed, Denmark) was used to identify the level of nitrogen of the sample (method 954.01). The nitrogen content was converted to crude protein by a conversion factor of 6.25. Van Soest et al. [14] was used to determine the NDF and ADF content. Neutral detergent fibre and ADF were expressed exclusive of residual ash. Neutral detergent fibre analysis did not include a heat-stable amylase. The lignin content of the sample was analysed by solubilisation of cellulose with sulphuric acid. Metabolizable energy was measured in rumen microbial inoculum using the in vitro gas production method. The in vitro gas production method ([15]) was used to prepare the buffer solution. Rumen fluid was obtained before morning feeding using a vacuum pump from three ruminally cannulated cows. The cows were fed grass hay (790 g/kg), wheat bran (203 g/kg), salt (3.2 g/kg), and a mineral and vitamin mixture (4.6 g/kg). Handling of the cows and rumen fluid sampling was approved by the Environmental and Occupational Health and Safety unit of International Livestock Research Institute. Fluids were composited (1:1, *v*/*v*), filtered through four layers of cheesecloth, and added to the buffer solution (1:2, *v*/*v*), which was kept in a water bath at 39 °C under continuous CO2 flushing. The buffered rumen fluid (30 mL) was transferred into 100 mL syringes containing 0.2 g of straw sample and immediately placed into a water bath at 39 °C. The 24 h gas production was recorded and used to calculate ME according to Menke and Steingass [16]. Phosphorous, calcium, magnesium, manganese, iron, and zinc contents were determined by an atomic absorption spectrophotometer (A. Analyst 300, Perkin Elmer, Shelton, CT, USA).

### 2.4. Statistical Analysis

The spectral data were not subjected to any mathematical treatment. The CENTRE algorithm was used to calculate the GH value (Mahalanobis global distance to the centre of the population) of each sample. Samples with GH ≥ 3 were considered as outliers. No outliers were found in the data; thus, all 190 samples were divided into two groups: a calibration group (160 samples) and a cross-validation group (30 samples). Multiple regression analysis was used to build the equations using the calibration group. The chemical composition of the validation set was predicted using the prediction equations and then the standard error of prediction was calculated (SEP). Calibration equations were evaluated using the coefficient of determination (R^2^), standard error of calibration (SEC), and SEP. Statistical analysis of the data was performed using WinISI II software.

## 3. Results

The means and standard deviation of the chemical composition, mineral analyses, and ME of chickpea and straw for both predicted and reference samples are presented in Table 2. There was wide variation in the chemical composition and ME of chickpea samples, which cover most of the variability reported in the literature.

### 3.1. Calibration

Results of the NIRS calibration are presented in Table 3. The R^2^c of the chickpea straw’s chemical composition ranged from 0.84 in DM to 0.99 in ADL. The R^2^c of Me was high (0.99). R^2^c Zn, Ca, Fe, Mn Mg, and P ranged from 0.71 to 0.93. The SEC of the proximate analysis ranged from 0.19% in DM to 0.85% in NDF. The SEC of ME was relatively small (0.06 MJ/kg DM) and the SEC of the mineral composition of chickpea straw ranged between 0.33% and 1.92%.

### 3.2. Validation

The results of the NIRS equations validation are shown in Table 3. R^2^v of the CP, NDF, ADF, ADL, and ME was higher than 0.96. Although, DM had a lower R^2^v of 0.78. The mineral composition of chickpea straw had a high R^2^v ranging between 0.68 and 0.92. The SEP of the proximate analysis and ME of chickpea straw ranged from 0.036% to 1.3%. The SEP of the mineral composition of chickpea straw Zn, Ca, Mg, Mn, Fe, and P was less than 3%.

## 4. Discussion

Improved varieties of chickpea produce significantly higher yields of grains compared to the local genotypes. They have high tolerance to drought and disease [17]. Thus, adopting these varieties would enhance the food security of developing countries and improve the livelihoods of farmers relying on chickpea production. National and international research centres have recognised that many improved varieties of crops are rejected by farmers in mixed farming systems due to the low palatability and nutritive value of straw [18,19]. This is a huge threat to the agricultural production and food security of a large number of households in mixed farming systems. Accordingly, straw’s nutritive value as a livestock feed became a priority in crop breeding programs to raise the rate of adoption of the improved varieties on the one hand and to enhance the nutritive value of the straw for livestock feeding on the other hand. Chickpea breeding programs generate huge sets of samples requiring screening for nutritive value in a short time [7]. Under such a scenario, conventional lab methods are not feasible as they are expensive, time consuming, and environmentally destructive. Here, NIRS offers a cheap, fast, and reliable method to accurately determine the nutritive value of a range of animal feed [20]. Furthermore, NIRS technology for feed analysis does not include dealing with any chemicals and does not have any animal welfare issue related to ruminal cannulation.

R^2^c and R^2^v of the chemical composition of chickpea straw were 0.78 for DM and higher than 0.96 for ash, CP, NDF, ADF, and ADL. This indicates a high match between the NIRS spectral data and conventional lab data of DM, ash, CP, NDF, ADF, and ADL of chickpea samples. In addition to this, SEC and SEP of NIRS equations for proximate analysis and cell wall constituents of chickpea straw were lower than 1%. Thus, these NIRS equations could predict the chemical composition of chickpea straw using spectral data. This is in agreement with previous studies, which reported that NIRS is an accurate method to predict the nutritive value of a range of animal feed [21,22,23,24,25,26].

R^2^c and R^2^v of ME of chickpea straw were high (0.99), indicating a strong correlation between the spectral data and in vitro ME data. The NIRS prediction equation of ME had low SEC and SEP (<0.1%). Therefore, the NIRS model produced from the current study is accurate in determining ME of chickpea straw samples. This is in agreement with results in the literature, which reported on the possibility of predicting some biological parameters including digestibility and ruminal gas production of feed using their spectral data ([22,26], respectively). Theoretically, the mineral composition of feed is not detectable by NIRS because their structure does not have organic bonds. However, minerals can be predicted if they are included in organic complexes [27] or due to the change that minerals cause in the water region of the spectrum [27,28,29]. The mineral composition of chickpea straw included in the current study had high R^2^c and R^2^v. Accordingly, the association between the spectral data and mineral composition of chickpea straw is high. Furthermore, both SEC and SEP were low (<1%). Therefore, the mineral composition of chickpea straw could be accurately predicted via the NIRS models generated by the current study. This is in disagreement with Goi et al.’s [30] study on the mineral composition of dog food, where Ca, P, Na, and Mg were poorly predicted using the NIRS equations. On the contrary, K and Na were accurately predicted using NIRS data [30]. Furthermore, minerals were predicted from NIRS data with low error in cheese (Ca, P, S, Mg, Zn, and Cu [31]; Na and K [32]), meat (Ca and Zn [33]), and processed meat products (Na [34,35,36]). This could be because most of the minerals in chickpea straw might be linked to oxalate in a form of oxalate salts or they are bound to other organic compounds. Near infrared reflectance spectroscopy could be used to facilitate chickpea breeding programs for food-feed improvement by offering an accurate, cheap, and fast tool for nutritional value determination.

## 5. Conclusions

Stationary NIRS is a feasible replacement technology to the conventional nutritional analyses to determine the chemical composition, ME, and mineral content (Zn, Ca, Mg, Mn, Fe, and P) of chickpea straw. This would decrease the cost (as it requires only technician time and cost of sample processing) and time of the analysis and environmental hazard associated with the conventional nutritional analysis methods. Chickpea breeders can screen chickpea genotypes for straw nutritive value using NIRS when chickpea programs target improving food and feed traits. These programs would release chickpea genotypes that are superior in grain yield and straw nutritive value, leading to more sustainable food production and food security in mixed farming systems.

## Figures and Tables

**Table 1:** Description of the experimental sites.

Characteristics	Locations				
	Akaki	Alem Tena	Chefe Donsa	Debre-Zeit	Minjar
Altitude (masl)	2200	1575	2450	1900	1810
Latitude	08°53	08°18	08°57	08°44	08°55
Longitude	38°49	38°57	39°06	38°58	39°45
Max temperature	26.5	29.8	26	28.3	28
Min temperature	7	12.9	7	8.9	10
Rainfall (mm)	1025	728	843	851	867
Rainfall distribution	Bimodal	Erratic	Bimodal	Bimodal	bimodal
Soil type	Vertisols	Light	Vertisols	Vertisols	Light
n of Desi genotypes	14	13	14	14	0
n of Kabuli genotypes	13	0	13	26	13
n of genotypes	27	13	27	40	13
Sn of samples	108	52	108	160	52

**Table 2:** Characterization of the data sets used to generate the prediction equation of chickpea straw.

Nutritional Composition	Reference	SD	NIRS	SD
	Mean		Mean	
DM (%)	90.40	0.47	90.41	0.39
Ash (%)	9.01	2.02	9.01	1.93
CP (%)	6.04	3.44	6.01	3.39
NDF (%)	53.75	8.64	53.79	8.6
ADF (%)	39.66	7.03	39.6	6.97
ADL (%)	9.13	1.82	9.13	1.77
ME (MJ/Kg DM)	7.86	0.88	7.88	0.87
Mineral composition				
Zn (mg/kg)	12.87	7.41	12.98	6.95
Mn (mg/kg)	70.54	44.4	70.11	42.1
Ca (g/kg)	13.52	3.72	13.47	3.29
Mg (g/kg)	2.01	0.43	2.02	0.39
Fe (mg/kg)	685	741	675	691
P (g/kg)	0.89	0.71	0.82	0.54

SD: standard deviation; DM: dry matter; CP: crude protein, NDF: neutral detergent fibre, ADF: acid detergent fibre; ADL: Acid detergent lignin; ME: in vitro gas production metabolizable energy.

**Table 3:** Values obtained from the NIRS calibration and validation of chickpea straw.

Nutritional Composition	Calibration	SEC (%)	Validation	SEP (%)
	R^2^c		R^2^v	
DM	0.84	0.19	0.78	0.24
Ash	0.97	0.35	0.96	0.56
CP	0.99	0.21	0.99	0.425
NDF	0.99	0.85	0.99	1.3
ADF	0.99	0.64	0.99	1.09
ADL	0.99	0.22	0.98	0.36
ME	0.99	0.06	0.99	0.036
Mineral composition				
Zn	0.93	1.89	0.91	2.27
Mn	0.89	1.2	0.89	1.46
Ca	0.91	1.07	0.89	1.66
Mg	0.88	0.15	0.84	0.16
Fe	0.92	1.92	0.92	2.08
P	0.71	0.33	0.68	0.45

R^2^c: coefficient of determination of calibration; SEC: standard error of calibration; R^2^v: coefficient of determination of validation; SEP: standard error of prediction; DM: dry matter; CP: crude protein, NDF: neutral detergent fibre; ADF: acid detergent fibre; ADL: Acid detergent lignin; ME: in vitro gas production metabolizable energy.

## Data Availability

The data presented in this study are available on reasonable request from the corresponding author.
